# Work-Based Assessments in Higher General Surgical Training Program: A Mixed Methods Study Exploring Trainers' and Trainees' Views and Experiences

**DOI:** 10.1055/s-0040-1708062

**Published:** 2020-03-09

**Authors:** Kamal Raj Aryal, Chelise Currow, Sarah Downey, Raaj Praseedom, Alexander Seager

**Affiliations:** 1 Department of General Surgery, James Paget University Hospital, Great Yarmouth, United Kingdom; 2 Department of General Surgery, University of East Anglia, Norwich, United Kingdom; 3 Department of General Surgery, Luton and Dunstable University Hospital, Luton, United Kingdom; 4 Department of General Surgery, James Paget University Hospital, Great Yarmouth United Kingdom; 5 Department of Hepatobiliary Surgery, Addenbrookes Hospital, Cambridge, United Kingdom; 6 Department of General Surgery, Peterborough City Hospital, Peterborough, United Kingdom

**Keywords:** work-based assessments, surgery training, procedure-based assessments, case-based discussion, clinical evaluation exercises, direct observation of procedural skills

## Abstract

**Introduction** In the United Kingdom, work-based assessments (WBAs) including procedure-based assessments (PBAs), case-based discussions (CBDs), clinical evaluation exercises (CEXs), and direct observation of procedural skills (DOPS) have been used in Higher General Surgical Training Program (HGSTP) since the introduction of Modernising Medical Careers. Although the Intercollegiate Surgical Curriculum Project states that they should be used for the formative development of trainees using feedback and reflection, there is no study to look at the perception of their usefulness and barriers in using them, particularly in HGSTP. The aim of this study is to investigate trainer's and trainee's perception of their usefulness, barriers in using them, and way forward for their improvement in HGSTP.

**Methods** This was a mixed method study. In phase I, after ethics committee approval, an online survey was sent to 83 trainers and 104 trainees, with a response rate of 33 and 37%, respectively, using Online Surveys (formerly Bristol Online Survey) from July 2018 to December 2018. After analysis of this result, in phase II, semistructured interviews were conducted with five trainees and five trainers who had volunteered to take part from phase I. Thematic analysis was performed to develop overarching themes.

**Results** For professional formative development, 15% of the trainers and 53% of the trainees felt that WBAs had a low value. Among 4 WBAs—CEX, CBD, PBA, and DOPS—PBA was thought to be the most useful WBA by 52% trainers and 74% trainees.

More trainers than trainees felt that it was being used as a formative tool (33 vs. 16%). The total number of WBAs thought to be required was between 20 and 40 per year, with 46% of the trainers and 53% of the trainees preferring these numbers.

The thematic analysis generated four themes with subthemes in each: theme 1, “factors affecting usefulness,” including the mode of validation, trainer/trainee engagement, and time spent in validating; theme 2, “doubt on utility” due to doubt on validity and being used as a tick-box exercise; theme 3, “pitfalls/difficulties” due to lack of time to validate, late validation, e-mail rather than face-to-face validation, trainer and trainee behavior, variability in feedback given, and emphasis on number than quality; and theme 4, “improvement strategies.”

**Conclusions** The WBAs are not being used in a way they are supposed to be used. The perception of educational impact (Kirkpatrick levels 1 and 2) by trainers was more optimistic than by trainees. Improvements can be made by giving/finding more time, trainer training, more face-to-face validation, and better trainer trainee interactions.

Several changes in postgraduate medical education and training including the European Working Directive, Modernising Medical Careers, and Postgraduate Medical Education and Training Board have shaped the current training structure of surgical training in the United Kingdom. 1 There has been a strong emphasis on assessing trainees in action (at the workplace). 2 Workplace-based assessment (WBA) is an “assessment of what doctors do in practice.” 3

In the United Kingdom, to become a consultant general surgeon, the doctors need to go through a lengthy training pathway, which includes a 2-year foundation year training program, a 2-year generic core surgical training program, and a 6-year higher surgical training program. 4 Several institutions are responsible for providing training in surgery. The Royal College of Surgeons through the Joint Committee on Surgical Training and its 10 Specialty Advisory Committees (SACs), for example, General Surgery SAC for general surgery, set up the curriculum standards for general surgery. Schools of Surgery at deanery level and Hospital Trusts at the local level run General Medical Council approved training programs. The curriculum delivered through the Intercollegiate Surgical Curriculum Project (ISCP) lays strong emphasis on specialty knowledge, clinical exposure, technical and operative skills, and professional skills and behavior.

The workplace for higher general surgery training may be different and unique to many other specialties. The higher general surgical trainee (HGST) sees and treats patients in the ward as inpatients, sees patients in the outpatient clinics, performs operations to treat several conditions, and looks after them both before and after the operation. The skills required to be developed for the completion of training are different from other specialties, particularly operative skills. The HGSTP is also a transition from core surgery training (basic surgery training) to becoming an independent practitioner as a consultant. In the HGSTP, the trainee practices independently in many areas where the trainee is already competent to perform tasks at workplace, whereas many actions and surgical procedures still require consultant supervision.

The WBAs include procedure-based assessments (PBA), clinical evaluation exercise (CEX), case-based discussion (CBD), direct observation of procedural skills (DOPS), and multisource feedback (MSF) to achieve skills of “performance” at the workplace. The WBAs used by this grade need to take into consideration to include all types of work they do in their place of work and need to fulfill all criteria as stipulated in the van der Vleuten utility formula. 5 6 MSF has been used for many years in many specialties and has been shown to help trainees to develop. 7 We will not be able to study MSF in this paper.

The literature search performed in December 2018 using Medline database yielded 17 surgical studies describing the usefulness of different WBAs (PBA, CEX, CBD, DOPS). Only eight studies purely on general surgery have included either trainee or trainer data only. 8 9 10 11 12 13 14 15 Most of these studies are on PBA. All except one have assessed educational impact only. There are not much data on CBD, mini–CEX, and DOPS. Also, basic trainees and higher trainees are included together in four of these studies. 8 9 13 14

Drawing these all together, though WBAs have been in use in HGST for nearly 10 years, gaps still remain in trainee and trainer experiences and the perception of the usefulness of WBAs in HGSTP, which this study will address.

Specific and achievable research questions (RQs) are as follows:

- RQ1: what is the trainee's and trainer's perception of the usefulness of WBAs for their learning and development?
- RQ2: what are the difficulties in using WBAs?
- RQ3: what are the ways of improving WBAs for better learning?

## Materials and Methods

### Theoretical Underpinning

It is important to have the views of both trainers and trainees who use these WBAs in their practice if we want to know about their usefulness, barriers of using them, and way forward for improvement. To answer these RQs, we chose a pragmatic approach on the basis that this research requires both quantitative and qualitative approaches. The quantitative phase has been guided by objectivism epistemology, meaning that there is only one reality, particularly around how the WBAs are practiced. Therefore, positivism, where scientific method produces precise verifiable answers and the fact can be revealed or discovered through the use of scientific method, is the theoretical perspective. 16 To achieve this, a questionnaire has been used as the method. 17 The qualitative part is guided by subjective ontology since different participants have different experiences and perceptions about WBAs. The underlying theory for this has a constructionism epistemology 18 and an interpretivism theoretical perspective. This means that there are multiple ways of knowing and that knowing is subjective. 19 The participants tell personal accounts and stories of their experiences, and we extract meaning on the content, context, structure, and relational aspects of the story. Individual semistructured interviews were used as methods.

This approach of mixed methods research has been found to have several advantages: increases construct validity to inform development of one method from other using methods sequentially, complements each other for enhancing and elaborating or for illustrating or clarifying results; triangulates to cross-check and corroborate results, and expands the range or scope of inquiry. 20 This was a sequential explanatory design: the questionnaire was followed by individual semistructured interviews. In this design, results or questions arising from quantitative data are explored qualitatively, which produce data that complements or clarifies the original findings. 20

Phase I includes mostly questionnaire survey. Some free-text comments such as “difficulties in using WBAs” and “improvements for each type of WBAs” helped to get their subjective view also. Phase II includes qualitative individual semistructured interviews with participants who agreed to take part in the initial survey and provided an opportunity to explore the results from phase I in detail.

### Phase I

The population was HGSTs from specialty trainee year 3 to year 8 (ST3 to ST8) and consultant surgeon trainers in the United Kingdom.

General surgical trainees from ST3 to ST8 from East of England Deanery and North Western Deanery were chosen as these were convenient to the author due to his place of training and place of work. For the pilot phase, participants were chosen from the author's professional network from other deaneries in the United Kingdom. The General Surgery Training Program Directors of these two deaneries asked the deanery administrative staff to send out the survey to HGST. For trainers, general surgery consultant trainer e-mail details were obtained with permission from the Regional Representative of East of England Association of Coloproctology of Great Britain and Ireland. Then the author sent out the survey to those trainers. Manchester-based Doctors Academy helped in sending out the survey to consultant trainers across North West of England.

The questionnaire was sent using Online Surveys (formerly Bristol Online Survey). There was a pilot survey in four trainees and three trainers before sending out the survey on a wider scale. Since the questionnaire has been designed taking into consideration the findings of previous papers on the subject 21 22 and PBA questionnaire published in another paper, 23 formal reliability and validity testing of the instrument was not performed. This was done to assess the feasibility of the study, and feedback was requested about the questions asked and if anything needed changing. Based on the responses received from these seven participants in the pilot phase, there was no need to change the content of the questions, although some of the phrasing and grammar was changed for the online survey. The final survey was sent to all eligible 100 trainees and 80 trainers in total, excluding the pilot phase. Two reminders were sent for the response. The survey questions included a combination of Likert scale questions, yes/no questions, categorical questions (on PBA), and open-ended free-text comment questions ( Supplementary Material 1 ; online only).

Data were extracted from the Online Surveys and entered into Microsoft Excel spreadsheet for analysis. Due to the small response size, the data were analyzed descriptively and are presented as frequencies and percentages to aid visual comparison. The participants who took part in the pilot phase are trainees and trainers with similar grade and experience as those from the main survey. None of the participants who took part in the pilot phase took part in the main survey. Therefore, the pilot survey responses have been combined with the main survey. Trainee and trainer responses were compared where possible, as were responses on different WBAs. Thematic content analysis was used to analyze the free-text/qualitative data. This helped to design semistructured interview questions for the phase II qualitative study. 24

### Phase II

Qualitative individual semistructured interviews were used to understand the perception and impact of WBAs in trainers and trainees.

All participants in phase I were asked if they would like to take part in phase II. If so, they were asked to provide their contact e-mails to arrange participation in phase II of the study. Those who volunteered to take part were contacted through e-mail and interview arrangements were made. The Online Survey was designed and set up to maintain anonymity, and in view of this, none of the e-mail addresses of those who volunteered was linked to the online survey responses.

The aim was representation from trainees at all levels of seniority from ST3 to ST8, and experiences varied among the trainers from junior to senior levels. Although the sample size for qualitative interview was small, there were participants spread along the dimensions of trainer/trainee, years of experience, and so on. When no themes were emerging (saturated) from the interviews, further recruitment of new participants was not continued for phase II. 25

After written consent, semistructured interviews were conducted in April 2019 either face-to-face or by telephone using recording line software and then transcribed using verbatim transcription 26 by K.A. The trainer and trainee questions for semistructured interviews are presented in Supplementary Material 2 (online only). Involvement of the researcher as an insider in all steps provided an opportunity to familiarize with the data right from the beginning. Microsoft Word was used to transcribe the interviews. Thematic analysis was then performed, which included familiarization with the data by reading and rereading by the author as the first step. An inductive approach was used, meaning themes extracted fit in with data themselves rather than focus on specific RQs. 27 The data were then organized in a meaningful and systematic way by coding. The codes with similar messages were charted together, which led to generation of categories and overarching themes. 28 29 Microsoft Excel was used to chart the qualitative data code. Themes were reviewed to generate a thematic map. Generated themes, subthemes with definitions, and accompanying quotes to support these have been presented in the Results/Findings section. Ethics approval was granted by the University of Dundee Ethics Committee.

## Results/Findings

### Phase I: Online Survey

Altogether, 27 trainers and 38 trainees (total number 65) responded to the survey, with a response rate of 33 and 37%, respectively.

### Usefulness of Work-Based Assessments

**Fig. 1:**
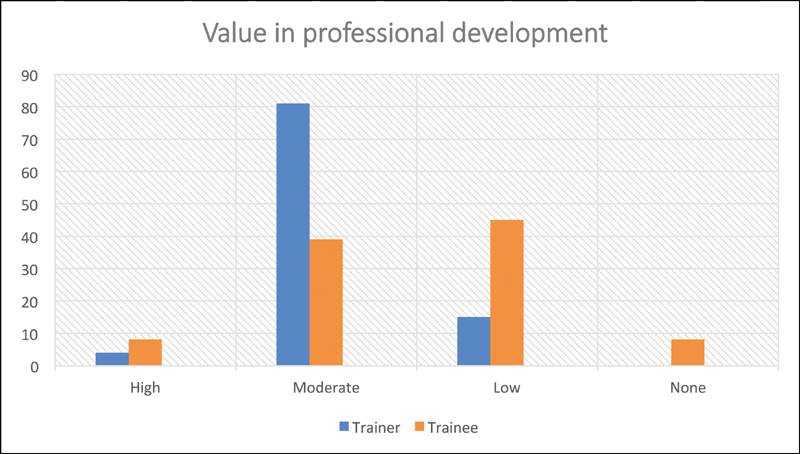
Value in professional development. WBA, work-based assessments.

**Fig. 2:**
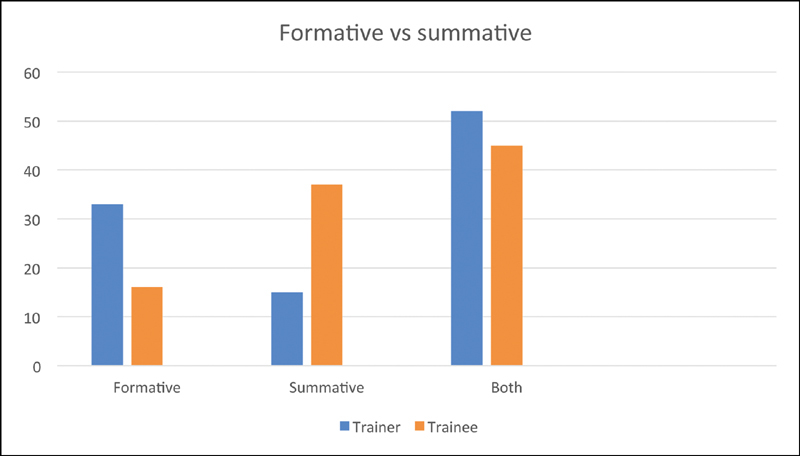
Formative or summative.

**Fig. 3:**
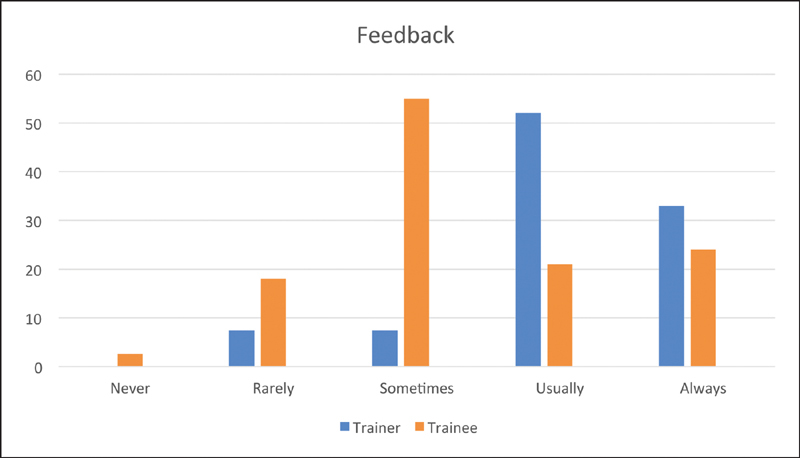
Feedback. WBA, work-based assessments.

**Fig. 4:**
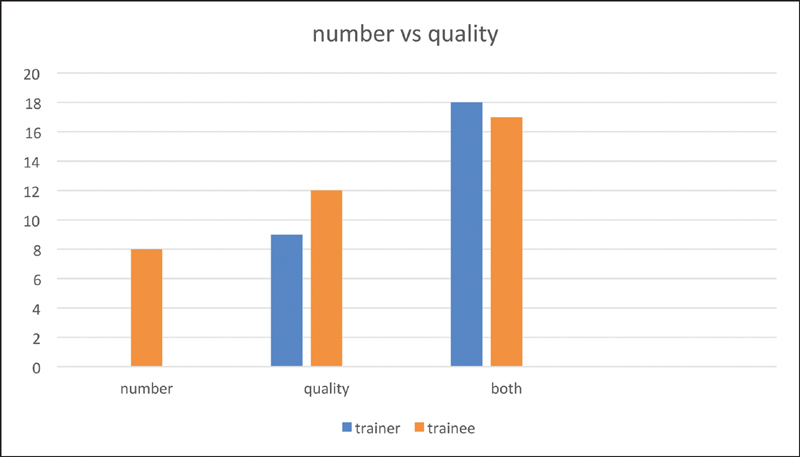
WBA number versus quality. WBA, work-based assessments.

**Fig. 5:**
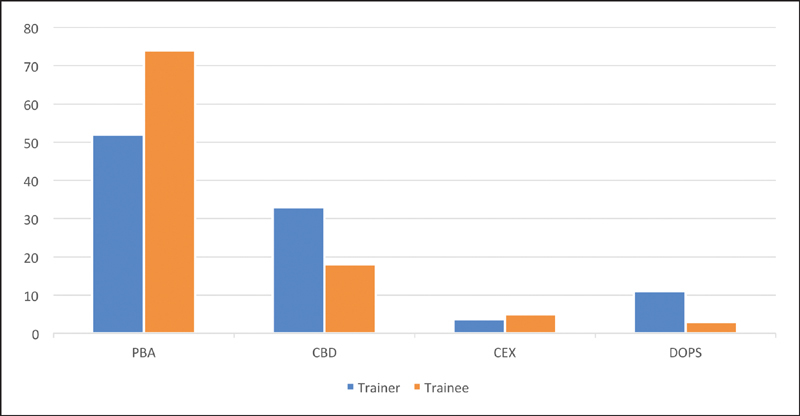
Most useful WBA. CBD, case-based discussions; CEX, clinical evaluation exercises; DOPS, direct observation of procedural skills; PBA, procedure-based assessments; WBA, work-based assessments.

The questions relating to perceived usefulness with questions by trainers and trainees are presented in Figs. 1 to 5 . Table 1 shows the total number of WBAs thought to be required by trainers and trainees, respectively.

**Table 1:** Number of WBAs per year: in your opinion, what is the ideal total number of WBAs required for each trainee per year?

	Trainer	Trainee
20 or less	9	8
>20 to 30	5	12
>30 to 40	8	8
>-40 to 50	3	7
>50 to 60	1	1
>60	1	2

Abbreviation: WBA, work-based assessments.

### Procedure-Based Assessment

**Fig. 6:**
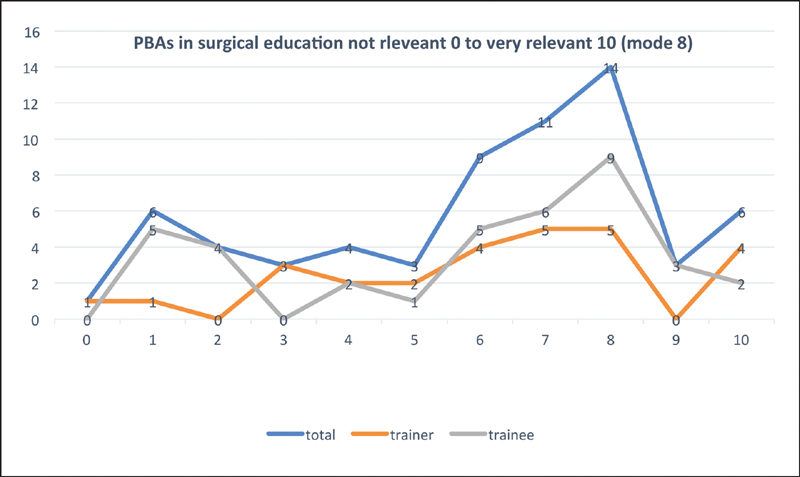
Relevance of PBA in surgical education. PBA, procedure-based assessments.

**Fig. 7:**
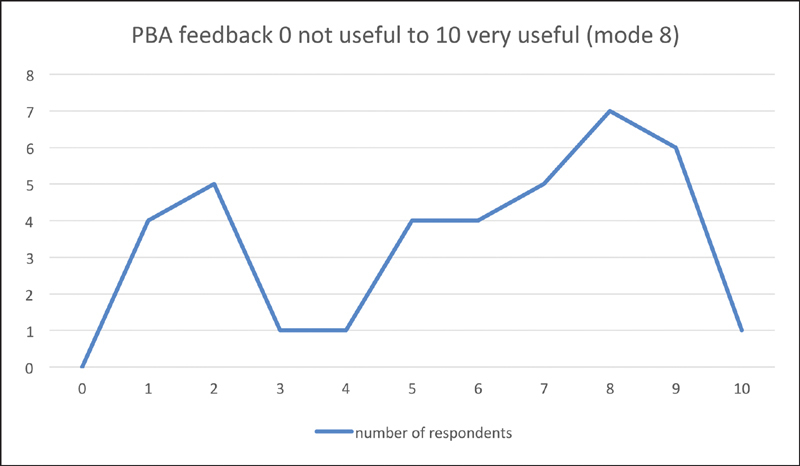
PBA feedback usefulness. PBA, procedure-based assessments.

For surgical education (0 = not relevant; 10 = very relevant), PBA had median score of 7 for combined trainer and trainee data (interquartile range 4–8), 7 for trainers (interquartile range: 4–8), and 6.5 (interquartile range: 2–8) for trainees ( Fig. 6 ). Similarly, median usefulness score on the feedback given by their trainers was 6 (interquartile range: 4–7) on a scale of 0 to 10 ( Fig. 7 ).

The thematic analysis of comment question results helped to generate questions used in semistructured interviews presented in Supplementary Appendix 2. To determine overarching themes, the qualitative data from phase I have been combined with the data from phase II.

### Phase II: Qualitative Study

This section describes details of the participants and different themes emerging from the interview. There were 10 participants whose details are shown in Table 2 .

**Table 2:** Details of participants

	Trainer ( *n* = 5)	Trainee ( *n* = 5)
Experience	Consultant time since appointment	
	3 years: 1	ST4: 1
	9 years: 1	ST5: 2
	10 years or more: 3	ST7: 1
	All: ES, CS	ST8: 1
Duration of interview (minutes)	17	18
	22	22
	24	33
	26	40
	47 (median: 24 min)	50 (median: 33 min)
Deanery	North Western: 1	London: 1
	East of England: 4	East of England: 4
Mode of interview	Face-to-face: 2 Telephone: 3	All telephone

Abbreviations: CS, clinical supervisor; ES, educational supervisor; ST, specialty trainee.

The thematic analysis of the comments of online survey and semistructured interviews generated three themes: two themes around RQ1 called “factors affecting usefulness” and “doubt on utility”; one theme around RQ2 “pitfalls”; and one theme around RQ3 “improvement strategies.”

The results relating to how WBAs are practiced in HGSTP currently is being published in another journal. 30 In summary face-to-face validation of WBAs took place in only 22% of trainer's view and 3% of trainee's view. A total of 26% of the trainers and 45% of the trainees said that e-mail without face-to-face contact was used for validation; 44% of the trainers and 92% of the trainees felt they had to send e-mail reminders for validation. Also, 33% of the trainers and 84% of the trainees felt that CEX validation took place without watching the trainee taking part in the event. Two themes emerged from the qualitative analysis of this paper: “more common to less common WBAs” and “method of validation.” The WBAs were not being practiced in a way they are supposed to be used in HGSTP.

### Usefulness of Work-Based Assessment

In summary, PBA was thought to be most useful for covering range of procedures, benchmarking, providing global summary, and monitoring progress and improvement. CBD was thought to be second most useful for covering topics in syllabus and is practical and relevant to use.

Overall, WBAs were thought to be useful because they provided learning opportunity, enhancement of knowledge, curriculum coverage, formalization of training, reflection tool, feedback tool, and adjuncts to educational supervisor (ES) and clinical supervisor (CS) report, but there were several factors affecting usefulness.

The reason for the unpopularity of DOPS and CEX was that surgical trainees have independence and it's very difficult to get the trainer to watch the trainee performing these tasks due to lack of time with service provision. The comments included that mini-CEX would be useful for leading a ward round observation, outpatient observation, breaking bad news, or consent for operative procedures.

### Theme 1: Factors Affecting Usefulness

Usefulness appeared to depend on how the WBA is validated/practiced (mode of validation and timing of validation) and on the engagement of trainee and trainer and trainee and trainer being on the same page. One participant said,

…when they complete it just based on the email again I am never sure if they really remember which case I am talking about even though I have put awful detail in. (Trainee ST4)

The most common mode of validation has been shown to be e-mail, and, unfortunately, as is evident from this quote, this mode is not found to be useful for the trainees.

Another participant commented about the timing of validation, stating that it is most useful if it is validated immediately after the event has taken place, as is evident from the following quote:

well it depends, I think they are useful if we do them there and then – I think that's the most useful time. Because you could pass judgement is fresh in your mind fresh in their mind… (Trainer 4)

From trainee's perspective, the engagement of the trainer determines the usefulness of the WBAs, as we can see from the following quote:

I feel it is very much a tick box exercise if you ask my opinion uhm and I think as I said before it very much depends upon the engagement of the person completing it, the trainer. (Trainee ST5)

### Theme 2: Doubt on Usefulness

There was some doubt on the usefulness due to doubt on validity (elaborated on pitfalls), with WBAs being used as a tick-box activity, doubt on actual value to trainee in current practice, and need for new methods of assessments called general professional capabilities (GPCs) or entrustable professional activities (EPAs), which will come to effect soon in the training system.

One participant said, *“* I feel there is an inherent bias in the system because a lot people send assessments say about what went well without sending the ones which did not go well” (trainee 3, ST4). This means that all learning events are not recorded, decreasing the content validity of the instrument.

The other reason for doubt in viability is because they are used as a tick-box exercise, as is evident from the following quotes:

I don't find them that useful because they are very time consuming very elaborate format you know clicking through these things, they are just the tick box exercise. (Trainee 2, ST5)

However, in my view a lot of time this ends up as a tick box exercise and number crunching exercise with no real value in the sense that not quite sure what the trainees get out of it. (Trainer 5)

### Theme 3: Difficulties/Pitfalls in Using Work-Based Assessments

The difficulties identified from the interview, with few important quotes, are shown in Table 3 . This included lack of time to validate, late timing of validation, e-mail rather than face-to-face validation, variable quality of feedback, and lack of follow-up on feedback, tick-box exercise, importance in number than quality, and loss of accuracy.

**Table 3:** Difficulties in using WBAs

Subthemes	Categories	Definition	Selected supporting quotes
Lack of time	Lack of time, job plan	Lack of time to validate	“I think the principle of sitting down and discussing in depth, in detail – a case is very good idea…once a week where the registrar brings a case where you sit down and discuss it. I never do that, and I don't have time to dedicate to that, I don't have that job planned, I don't have that available” (trainer 2)
	Time-consuming	Time required to validate	“Certainly, in my current job it would be very difficult to get the consultant to sit down with you and do these things nearly impossible. Uhm and also it's time consuming for everyone” (trainee 2, ST5)
Timing of validation	Late sending	E-mail sent late following the event	“But equally if they send you from 3 months later there is absolutely no desire to do it because you don't remember it…” (trainer 4)
	No time limit	No time limit on the time from event to validation at present	“There is no restriction, they must do with in a certain time frame, and I think they are a bit more relaxed about it” (trainer 4)
	Not sent uniformly	Sent for validation in blocks rather than regular intervals	”Exactly and then try to do with on a regular basis but I do struggle to do that so I end up having blocks when I do loads and then may be a month when I do not do anything and start again to do the block” (trainee 2, ST5)
Mode of validation e-mail	E-mail	Sending ticket by e-mail rather than face-to-face	“In my experience even from my core training to where I am now uhm I mean uhm the perception is that consultants prefer if you just send them the ticket and it's just faster, they just have to click” (trainee 2, ST5)
Feedback	Follow-up on feedback	Not possible to check if the feedback given has been acted on or not	“You might give feedback with developments, suggestions but you have no way of following that up” (Trainer 1) “To be honest, once it's signed off, I do not look at it always. yes (pause) (laughter)” (trainee 2, ST5)
	Quality of feedback	Quality of feedback variable	“All of time it is just continue (laughter). Has good experience. Continue that sort of thing or do more cases read more about it so a lot of time that's the feedback” (trainee 4, ST4)
Forms	Tick box (came in all interviews)	WBAs used as a tick–box exercise	“We do treat this as tick box exercise so the more you can do the better and less time is needed” (trainee 2, ST5)
	PBA forms long	Long forms to fill up	“…for the PBAs particularly, there's a list of 30 odd steps for each thing, which you end up just ticking boxes to complete the form” (trainer 2)
Quality	Set number than quality	There is a set required number to pass ARCP, which may be very high	“Number than quality. If you have 39 you do not pass your ARCP. So, the focus is really on volume and not a single time in ARCP there has been a question asked about the quality of the WBA, depths of my reflection or anything like that and it is numbers exercise” (trainee 1, ST8)
Validity	Loss of accuracy	Doubt on accuracy of validation	“The trainer will say yes send me a WBA that's fine and then despite numerous reminder you send through ISCP the trainer just won't complete them and then sort of 4–5 months down the line…and I feel by then the trainer probably forgotten that case or he is not in a good position to put comments for some time ago I think the accuracy of that assessment is not great” (trainee 5, ST7)

Abbreviations: ARCP, Annual Review of Competence Progression; ISCP, Intercollegiate Surgical Curriculum Project; PBA, procedure-based assessments; ST, specialty trainee; WBA, work-based assessments.

### Theme 4: Improvement

Based on the thematic analysis of the semistructured interview and online survey, improvement centered on trainer and trainee, making/finding time, timing of validation, mode of validation, forms, feedback, quality of WBAs, and lessons from other specialties. Most frequently occurring subthemes with supporting quotes are presented next.

#### More Trainer Training

I don't know very much about what training they actually do get in to in filling in those assessments and giving feedback. I am sure they get something. I think if we were on the same page, they will perhaps be little bit more aware of all the curriculum requirements and the number of assessments we need year on year. I think that may make it easier. (Trainee 3, ST4)

I think there needs to be carrot and stick approach. If you are going to get a trainee, you have to engage with them. (Trainer 4)

….not all of us are going to be trainers so people who are training should be recognised for their effort. (Trainer 4)

#### More Trainee Initiative

If they want to do one they need to tell me beforehand then you plan to do then. (Trainer 4)

#### Finding Time

Well (pause) if you are going to have a trainee you have to make time for them. It's not acceptable to say it's not part of the job plan or that's nonsense. You have to make time. (Trainer 4)

#### Timeline on E-Portfolio to See Where the Trainee Is in Completing WBAs

I think there needs to be a mechanism in place where both the trainer and trainee must realise that it's either done within a week or at most 10 days or it goes to pot. (Trainer 4)

When I am an AES, I always tell them try to do one a week, you should never have any problems. (Trainer 4)

#### Face-to-Face Mode of Validation Better

Had more sit-down time with your consultant have a proper debrief that would add so more value and add more comments on the boxes but that would have to be done at the same time. (Trainee 3, ST4)

#### Feedback Should Be Constructive

I am quite honest with my feedback in that I am not afraid to bad it's positive criticism - positive constructive criticism. (Trainer 5)

#### Forms to Fill Up for Validation Should Be Revisited

I think you can skip all those tick boxes and just do those comment boxes. It should be quick to do. It's time and conversation you had with the assessor rather than the click things. (Trainee 1, ST8)

#### Assessment of Quality of Work-Based Assessments

I suppose if there is a way to (pause) may be sample some of those WBAs at their ARCP looked at handful…that might be quite good because if people know that it is actually going to be looked at and people are going to talk about them then it means it's going to get more thorough. (Trainee 5, ST7)

**Fig. 8:**
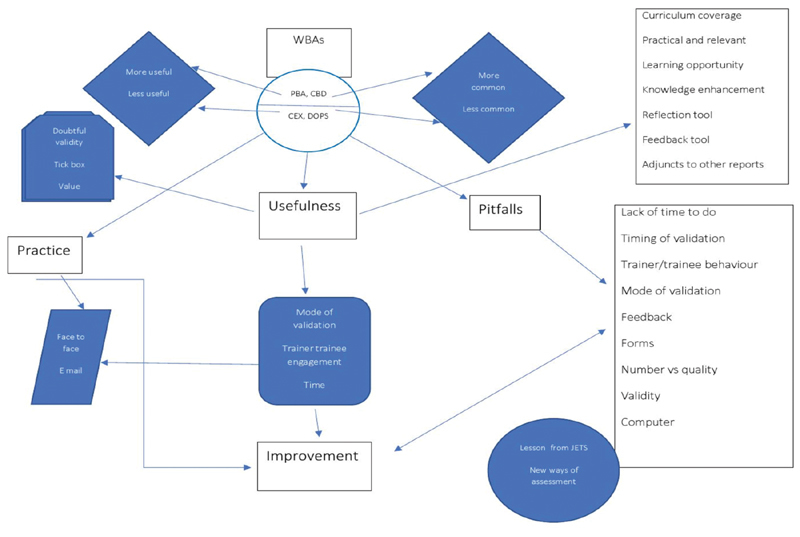
Thematic map. CBD, case-based discussions; CEX, clinical evaluation exercises; DOPS, direct observation of procedural skills; JETS, JAG Endoscopic Training System; PBA, procedure-based assessments; WBA, work-based assessments.

The thematic map is presented in Fig. 8 . As we can see among the four types of WBAs, more common to less common WBA correlated with more useful to less useful WBA. While, overall, they were thought to be useful, there were some doubts about utility, and this depended upon trainer, trainee engagement, finding time, and how they are practiced (mode of validation). Improvements can be made by acting on several pitfalls presented and understanding how they are practiced and used.

## Discussion

This study is the first study completed across the HGSTP incorporating both trainer and trainee views.

### Perceptions of Usefulness of Work-Based Assessment (RQ1)

The usefulness or utility of an assessment has been defined as a product of educational impact, validity, reliability, cost-effectiveness, acceptability, and practicality (feasibility). 5 Educational impact was assessed using Kirkpatrick's hierarchical model of evaluation pyramid from a lower level to a higher level: level 1, “satisfaction”; level 2, “learning”; level 3, “behavior”; level 4, “results.” 6 This study was able to assess only levels 1 and 2.

In this study, 15% of the trainers and 26% of the trainees felt that WBAs were poor assessment tools. These results were slightly more optimistic compared with another study using a survey in which 35% of trainees rated WBAs as poor assessment tools. 31 The ISCP guidance on using WBAs states the following: “the tools are not intended to score trainees or summate progress globally.” A previous study has shown that they are used as a summative tick-box exercise, 32 33 and in this study, 37% of the trainees felt that it was being used as a summative tool alone.

The perception of feedback being given never or rarely in 21% of trainees and 7.5% of the trainers in this study is better than the previous survey in orthopaedic practice, where 37% (trainees) and 13% (trainers) felt so. 34 Trainees also felt that they were upset with feedback given sometimes and usually (15 and 13%, respectively). This negative effect of feedback may relate to how the feedback was given. Feedback from WBAs, if well delivered, leads to a perceived positive effect on practice, as seen in another study. 7

While CEX was thought to be third most useful by trainees, trainers thought that DOPS was the third most useful. These results are similar to another study involving trainees, 15 but the popularity of DOPS and CEX was much less in this study compared with a previous study related to orthopaedics. 31

Perceived usefulness appeared to depend on how the WBA is validated, engagement of trainee and trainer, and trainee and trainer being on the same page. A previous study has highlighted individual and organizational factors that influence the usefulness of WBAs. 32 There was some doubt about the usefulness of WBAs due to doubts regarding validity, WBAs as a tick-box exercise, and possible lack of actual value to the trainees in current practice.

The total number of WBAs thought to be required was between 20 and 40, with PBAs accounting majority of those numbers in this study. Another study of trainers and trainees in orthopaedic surgery showed that the most favored number was 18 among a set of fixed numbers of 0, 6, 18, 40, and 80. Rather than a fixed number, a range of 20 to 40 may be more suitable. The numbers need to be such that they do not overburden trainees and trainers, which may lead this to WBAs as being used as a tick-box exercise, 33 whereas, on the other hand, the numbers need to be sufficient to maintain reliability. 34 35 In this study, 12 of 38 trainees and 9 of 27 trainers felt that quality was more important than quantity required at the Annual Review of Competence Progression (ARCP). Quality is not checked at ARCP at present. The trainer responses were seen more positive than the trainee responses in this study.

### RQ2 Barriers to Using Work-Based Assessments

Several barriers were identified in this study in WBAs, including lack of time to do, timing of validation (delayed validation causing forgetfulness and loss of accuracy), e-mail rather than face-to-face interaction, trainer trainee behavior including loss of engagement, emphasis in number than quality, poor feedback, problem with forms, doubt on validity, and computer issues. Previous studies found a lack of trainee and trainer's time, difficulty in finding a willing and suitable assessor, lack of enthusiasm, lack of trainer training and knowledge about trainee's requirements, the delay between event and completion of feedback, and poor understanding of the purpose of WBAs. 36 37 38 Our study has found several additional factors such as difficulties in e-mail validation, emphasis on number than quality, doubt on validity, and computer issues not noted in the previous studies.

Trainer/trainee behavior and feedback from the trainer if delivered and perceived positively help in learning. From this study, it is clear that the WBAs are being used to “look good” and using cases that went well rather than including cases that had complications or problems. In a previous qualitative study, trainees perceived the purpose of WBAs as “learning” or an “assessment of learning,” and the trainees chose an approach of “play the game” to seek positive feedback. 39 40

### The Way Forward for Improvement (RQ3)

Based on this study, the following recommendations can be made across areas of practice, trainer/trainee interaction, time, feedback, lessons from other specialties, and use of new ways of assessment. These are tied to the thematic analysis performed in semistructured interview and comments made in online survey.

- Trainer/trainee: there should be regular trainer training and education. This may also improve trainer belief in the system. Trainers should be given incentives and recognition. This system is likely to increase enthusiasm, motivation, and engagement. Trainer proactivity aids better learning for the trainee. Trainees should take a proactive role in WBAs and should communicate their intentions of completing WBAs well in advance to their trainers for better learning. They should be motivated trainees with dedication and use reflective practice. The trainee and trainer should be on the “same page” with a common goal of trainee's learning and development.

- Time (lack of time and timing of validation): lack of time is a great difficulty and can be addressed by better job planning, organization of time table including diarizing WBA sessions in the time table, and so on. Dedicated training lists like they do in endoscopy with an appropriate number of patients would help make some time to do WBAs face-to-face. The timing of validation should be immediately following the clinical encounter. In case of failing to do so, there should be a time limit within which the validation should take place. They should be performed uniformly over the training period rather than in blocks. There should be a time bar on the ISCP portfolio.

- Method of validation/feedback: face-to-face validation should be used more rather than only e-mail. Feedback using these sessions should be timely, personalised, constructive. There should be a plan to follow up on the feedback. Meeting up with CSs at regular intervals and entry of this in the ISCP portfolio which does not happen at present may be useful for overall feedback rather than WBA specific feedback.

- Forms and quality: the forms, particularly PBA, should be shorter and clearer. Emphasis should be given in comment boxes rather than tick boxes. The important aspects should appear at the top of the form rather than the bottom. The WBAs should be quality-checked either by an ES or ARCP panel by sampling a certain number of forms if it is not possible to check all of the forms. The total number required per year should be reduced to less than 40. PBA and CBD should be the main forms, but CEX consent and CEX for breaking bad news should be used more often. DOPS can be used if PBA is not available for a certain procedure, but there is a need for expansion of PBA forms to cover other common procedures rather than limiting them to few indexed procedures.

- Improving validity: attempts should be made to include overall learning events even if the outcome/experience was not good to increase the validity and learning episode of “on the job learning.” The reflections should not be used against trainees. The individual WBAs should not be used in a summative way.

- Innovations: lessons should be learned from other specialties including the JETS (JAG Endoscopic Training System) portfolio for endoscopy training. The training clinics with an appropriate number of patients like in general practitioner training clinics may be useful, particularly for junior registrars ST3 to ST5.

- New ways of assessments including EPAs and GPCS assessed by capabilities in practice and multiple consultant review (MCR) should be incorporated for professional and global skills, 41 42 and WBAs should be retained for the development of specific skills. Going through incomplete WBAs during times of MCR, which can be incorporated in monthly mortality and morbidity days, will help complete any incomplete WBAs for validation.

### Limitations of the Study

The number of invitations sent out for the online survey was less than planned. The disappointing response rate may have been because of the online survey being rather long with many questions. The time required to complete the questionnaire was approximately 15 minutes. This may have discouraged the candidates to take part. The number of participants for the semistructured interview was based on the volunteers who agreed to take part in the online survey, and there may have been bias in taking part by those who are interested. The number of the semistructured interviews performed was sufficient to reach the saturation point. It was not possible to get trainees and trainers together for focus group discussions due to their work commitments. Also, the number of participants who agreed to take part in interviews from online survey was felt to be too small to take part in focus group discussions. Although focus group discussion may have a larger number of participants, it is not always better than semistructured interviews because the participants who are less confident and quieter may not take part in the discussion and their confidentiality is not maintained as it is in semistructured interviews. 43

## Conclusions

The usefulness of WBAs depends on how they are used, trainee and trainer engagement, and being on the same page. Several barriers described here can be overcome by various means including better planning, face-to-face validation, trainer training, and using lessons from other specialties. Future larger-scale studies on WBAs including follow-up in feedback and newer ways of assessment are needed for improvement.
