# Supplementary material for: Work-Based Assessments in Higher General Surgical Training Program: A Mixed Methods Study Exploring Trainers' and Trainees' Views and Experiences
Source: Surg J (N Y). 2020 Mar 9;6(1):e49–61. doi: 10.1055/s-0040-1708062 (PMC7062550; doi:10.1055/s-0040-1708062)
Supplement: Supplementary file 1 — Supplementary Material [file 10-1055-s-0040-1708062-s1900077oa-1.pdf]

Work Based Assessments (WBAS) in Higher General Surgical Training Programme - Trainer Views and Way for improvement (copy)

Page 1: Page 1

Study Overview

Online survey Trainee - Study overview

Work Based Assessments in higher surgical training programme - trainer and trainee views and way for improvement

Invitation to take part in a research study

You are being asked to take part in a research study as part of Masters in Medical Education conducted by Kamal Aryal and supervised by Mandy Moffat which looks at Work Place Based Assessments (WBA). This includes your views on Procedure Based Assessments (PBA), Case Based Discussions (CBD), Clinical Evaluation Exercises (CEX) and Direct Observation of Procedural Skills (DOPS). We want to know your views using the questionnaire presented here.

Purpose of the study

The aim of this study is to explore the usefulness and impact of WBAs in higher general surgical training in the UK. We are trying to address following research questions:

- What is adequate number of WBAs required at each Annual Review of Completion of Training (ARCP) in trainee and trainer's view?
- What is the trainee and trainer's perception on impact and usefulness of WBAs for trainee's learning and development?
- In what ways can WBAs be improved to support learning?

The research is being conducted in two phases. This is the first phase using an online survey. The second phase involves structured focus group interviews. If you would like to participate on the second phase, please enter your e mail on the last page. This last page won't be visible during analysis to keep anonymity.

Participation in this research would benefit trainees in the improvement of WBAs in the long term.

Time commitment

This will take approximately 20 minutes to complete on computer.

Risks

There are no known risks for you in this study.

Termination of participation

You may decide to stop being a part of the research study at any time without explanation and without penalty.

Confidentiality/anonymity

The data collected do not contain any personal information about you.

No one will be able to link the data you provide to your identity and name.

The data will be seen only by the researchers and will not be made available to anyone else.

The questionnaires will be kept for 3 years after which time the files will be destroyed.

The results may be published in scientific meetings and conferences and may be published in peer reviewed journals. There will be no participant identifiable data in these presentations.

For further information about this research study

Kamal Aryal will be glad to answer your questions about this study at any time. You may contact him at kamal.aryal@jpaget.nhs.uk, telephone no 01493452232.

The University Research Ethics Committee of the University of Dundee has reviewed and approved this research study.

Kamal Aryal

kamal.aryal@jpaget.nhs.uk, telephone no 01493452232

Page 2: Background information

1. What is your Local Education Trust Board (LETB)/Deanery

2. What is your level of training?

- ST3
- ST4
- ST5
- ST6
- ST7
- ST8
- Out of programme

3. What is your Subspecialty?

- General
- Colorectal
- Vascular
- Upper GI (O and G)
- Breast
- Transplant
- Upper GI (HPB)

Page 3

WBAS in General

4. How often do you tell your trainer beforehand that you want to have WBA done before the session actually takes place?

- less than 25%
- more than 25% but less than 50%
- more than 50% but less than 75%
- More than 75%

5. How do you usually complete WBAs up?

- a. Face to face with trainee
- b. Face to face first then via email
- c. via email without (face to face?) discussion with trainee

6. Do you have to remind your trainer to do these if done by email? If yes go to next question if no skip next question.

- Yes
- No

6.a. If yes what percentage of WBAs performed need to be reminded?

- less than 25% of the WBAS performed
- More than 25% but less than 50%
- More than 50% but less than 75%
- More than 75%

7. In your opinion what is the most useful WBA for higher general surgical trainees?   Comments

- PBA
- CBD
- DOPS
- CEX

Please comment for your choice

8. Overall, how do you rate WBAS?

- Very good
- Good
- Average

- Poor
- Very poor

9. What is the minimum number of WBAS required in your deanery per year?

- 20
- 30
- 40
- 50
- 60 or more

10. In your opinion what is the ideal total number of WBAS required for each trainee each year?

- Less than 20
- 20-30
- 30- 40
- 40-50
- 50-60
- more than 60

11. What aspect of WBAS do they check at ARCP?

- Number
- Quality
- Both

12. What is their value in trainee's professional development? (formative)

- High
- Moderate
- Low
- None

13. In your experience, are they used as summative or formative?

- a. Formative
- b. Summative
- c. Both

14. How often do you complete/validate WBAs?

- Four times per week
- Three times per week
- Twice per week
- Once per week
- Once in 2 weeks
- Once in 4 weeks
- Once in 2 months
- Very haphazard

15. Do your trainers give you feedback (written/verbal) using WBAs?

- Never
- Rarely
- Sometimes
- Usually
- Always

16. Do you feel upset after you finish WBA if conducted face to face?

- Never
- Rarely
- Sometimes
- Usually
- Always

17. Who takes initiation in completing those?

- Trainer
- Trainee

18. Is there enough time allocated to perform these in your work?

- Yes
- No

19. What aspect of WBAS is it important to check at Annual Review of Competence Progression (ARCP)?

- Number
- Quality
- Both

20. What difficulties you have in validating WBAS? (please insert free text)

7 / 13

21. How could we improve WBAs? Your comment may be about the forms being used or about the process of WBA session (please insert free text)

Page 4: Procedure Based Assessments (PBA)

The following link takes you to an example of PBA form for you to have a look if you need while responding to survey questions

https://www.iscp.ac.uk/curriculum/surgical/assesment_pba_pdf.aspx?enc=A88UaRs98jqqzrYvusguaA==

22. In your view, how many PBAS are required for higher surgical trainees each year?

- less than 10
- 10 to 20
- more than 20

23. When is PBA debrief session generally conducted after the operative procedure?

- Immediately after
- Within a week
- After 1 week

24. How important do you think PBAs are in surgical education? (please tick a number on the scale)

Please don't select more than 1 answer(s) per row.

	0	1	2	3	4	5	6	7	8	9	10	
Iirrelevant	☐	☐	☐	☐	☐	☐	☐	☐	☐	☐	☐	Very important

25. How useful have you found the feedback given by your supervising consultant in these sessions? (please tick a number on the scale)

Please don't select more than 1 answer(s) per row.

	0	1	2	3	4	5	6	7	8	9	10	
Iirrelevant	☐	☐	☐	☐	☐	☐	☐	☐	☐	☐	☐	Very important

26. To what extent do you think PBAs enhanced your trainer’s ability to assess you? (please tick a number on the scale)

Please don't select more than 1 answer(s) per row.

	0	1	2	3	4	5	6	7	8	9	10	
Not at all	☐	☐	☐	☐	☐	☐	☐	☐	☐	☐	☐	Very much

27. How are PBA debrief sessions completed mostly?

- via e mail without discussion with trainee
- sitting with trainee face to face
- face to face first then e mail

28. Does your trainer watch you operating on that procedure before you get the PBA validated?

- Never
- Rarely
- Sometimes
- Usually
- Always

29. Do you tell your trainer beforehand that you would like to have PBA for the procedure?

- Yes
- No

30. Do you do the form if the operation did not go on well for the operation you were hoping to do PBA?

- Yes
- No

31. All WBA forms have a section on Trainer feedback including general, strengths, development needs, recommended actions. However, PBA has one added component - What was done well? Do you think this addition is actually important?

- Yes
- No

32. Please give your views on improving PBAs? This may relate to the PBA form itself, the PBA process or any other (please insert free text)

Page 5: Clinical evaluation exercise (CEX)

The following link takes you to the CEX form for you to have a look if you need while responding to survey questions

https://www.iscp.ac.uk/static/public/CEXJul2015.pdf

33. Is it valuable for trainees (ST5 and above) in Surgery?

- ○ Yes
- ○ No

34. What type of clinical encounters do you think should be assessed by this method in surgery?

- ☐ History taking
- ☐ Clinical examination
- ☐ Communication to patient/family including consent
- ☐ Breaking bad news
- ☐ Ward round
- ☐ Leading a clinic

35. Does your trainer watch your performance to grade how you performed?

- ○ Never
- ○ Rarely
- ○ Sometimes
- ○ Usually
- ○ Always

36. Please give your views on improving CEX? This may relate to the CEX form, the CEX process or any other (please insert free text)

Page 6: Case Based Discussion (CBD)

The following link takes you to the CBD form for you to have a look if you need while responding to survey questions

https://www.iscp.ac.uk/static/public/CBDJul2015.pdf

37. Are they useful for trainees ST5 and above?

- ◯ Yes
- ◯ No

38. What type of cases do you get discussed mostly for CBD in your experience?

- ☐ simple cases
- ☐ complex cases

39. Does your trainer give you enough time to present your reasoning skills during debrief sessions?

- ◯ Yes
- ◯ No

40. Do you think the gradings stated in the forms are true reflection of trainees competence?

- ◯ Yes
- ◯ NO

41. Please give your views on improving CBD. This may relate to the CBD form, the CBD debrief process or any other (please insert free text)

Page 7: Direct Observation of Procedural Skills (DOPS)

The following link takes you to the DOPS form for you to have a look if you need while responding to survey questions

https://www.iscp.ac.uk/static/public/DOPSJul2015.pdf

42. Are they useful for trainees ST3 and above?

- Yes
- No
- Not sure

42.a. If yes, what type of procedure would be useful for trainees for ST3 and above to be included in as DOPs?

- Endoscopic procedures
- Indexed operative procedures
- Others

42.a.i. If others please state option

43. For higher surgical trainee which one would you prefer better WBA for operative surgical procedures between DOPS and PBA.

- DOPS
- PBA

44. Please give your views on improving DOPS. This may relate to the DOPS form, the DOPS debrief process or any other (please insert free text)

Page 8

Thank you for completing this survey. Your responses have now been submitted

Focus Group

The second part of this study is qualitative focus group discussion and or semi structured individual interviews If you would like to take part in the second part of this study please follow to following link. This will take you to a second survey containing one question regarding your contact details (*for the purpose of the focus group*).

It also ensures your contact details are not linked with your responses to this completed survey for anonymity.

https://admin.onlinesurveys.ac.uk/account/dundee/survey/edit/358978

.

Key for selection options

1 - What is your Local Education Trust Board (LETB)/Deanery

- NHS Education for Scotland
- Northern Ireland Medical and Dental training agency
- Wales Deanery
- Health Education North East
- Health Education North West
- Health Education Yorkshire and Humber
- Health Education East Midlands
- Health Education West Midlands
- Health Education East of England
- Health Education Thames Valley
- Health Education Kent, Surrey and Sussex
- Health Education Wessex
- Health Education South West
- Health Education North Central and East London
- Health Education North West London
- Health Education South London
