# Supplementary material for: Work-Based Assessments in Higher General Surgical Training Program: A Mixed Methods Study Exploring Trainers' and Trainees' Views and Experiences
Source: Surg J (N Y). 2020 Mar 9;6(1):e49–61. doi: 10.1055/s-0040-1708062 (PMC7062550; doi:10.1055/s-0040-1708062)
Supplement: Supplementary file 2 — Supplementary Material [file 10-1055-s-0040-1708062-s1900077oa-2.pdf]

Supplementary Material 2: Semistructured interview questions

Trainer

Thank you very much for this interview.

- What is your experience in using WBAs? What type of WBAs have you had experience with?
- What is your perception of their *usefulness*?
- What *difficulties* have you had in using/validating them? Give me some examples.
- In your opinion, how can we *improve* them?

Opinion/probes on difficulties in using them if not covered during the above discussion (these themes have arisen following online survey)

-What about the time to do these?
-What about the timing of validation following the event?
-What about the mode of validation? E-mail or face-to-face?
-What about giving feedback written in forms versus constant feedback given during training?
-What about forms? Tick boxes? Granular?
-What about trainee's role? Proactivity? Trainee engagement?
-What about access to computer, passwords, and so on?
-What about curriculum coverage?

Opinion/probes on *improvements* if not covered during the above discussion (these themes have arisen following online survey)

- What about allocating enough time?
- How can we improve the timing of validation to make sure this is done early?
- What type of validation (interaction of validation) should we have? For example, face-to-face, e-mail?
- What about giving feedback?
- What about forms? Anything to improve?
- How can we improve quality?
- What about computer issues?
- What about training of faculty?

Any comments about improving individual WBAs: PBA, CBD, CEX, and DOPS?

Is there anything else you would like to share about WBAs from your experience?

Trainee

Thank you very much for this interview.

- What is your *experience* in using WBAs? What type of WBAs have you had experience with?
- What is your perception of their *usefulness*?
- What *difficulties* have you had in using them? Give me some examples.
- In your opinion, how can we *improve* them?

Opinion/probes on *difficulties* on using them if not covered above during discussion (these themes have arisen following online survey)

-What about time to do?
-What about the timing of validation following event and delay in validation?
-What about the availability of trainer to do?
-Is there any problem with feedback given?
-What about the method of validation any problems? For example, e-mails instead of face-to-face validation.
-Is there any difficulty with computer password issues?
-Do you have a breadth of curriculum covered using WBAs?
-What about trainee/trainer engagement? Why could that be due to?

Probes/opinion on *improvement* if not covered above during discussion (these themes have arisen following online survey)

-What about allocating time/session for validation?
-When should validation following completion of event take place? How can we make sure this happens early?
-Who should these be led by? trainer or trainee?
-How can we improve trainer and trainee engagement?
-How can we ensure the feedbacks given are more effective?
-How can we improve quality rather than number?
-What method of validation is better and how can we aspire toward that?
-How can we improve computer issues?
-How can we improve forms?

- Any comment on improving individual WBAs: PBA, CBD, CEX, and DOPS?
- Is there anything else you would like to share about WBAs from your experience?

Abbreviations: CBD, case-based discussions; CEX, clinical evaluation exercises; DOPS, direct observation of procedural skills; PBA, procedure-based assessments; WBA, work-based assessments.
